# 2-Aminoadipic Acid mediates the association between BMI and Lipids: Evidence from the CLSA and BHS Cohorts and Mendelian randomization

**DOI:** 10.1016/j.ajpc.2026.101560

**Published:** 2026-03-15

**Authors:** Ruiyuan Zhang, Yixi Sun, Yizhuo Liu, Tingting Liu, Yanling Qi, Lang Wu, Xiao Sun, Zhijie Huang, Yang Pan, Adrianna Westbrook, Shengxu Li, Lydia Bazzano, Wei Chen, Jing Chen, Jiang He, Tanika Kelly, Changwei Li

**Affiliations:** a O’Donnell School of Public Health, University of Texas Southwestern Medical Center, Dallas, TX, USA; b Division of Epidemiology and Biostatistics, School of Public Health, University of Illinois Chicago, Chicago, IL, USA; c Division of Endocrinology, Diabetes and Metabolism, Department of Medicine, College of Medicine, University of Illinois Chicago, Chicago, IL, USA; d College of Nursing, Florida State University, Tallahassee, FL, USA; e Department of Health Care Administration, California State University Long Beach, College of Health and Human Services, Long Beach, CA, USA; f Population Sciences in the Pacific Program, University of Hawai’i Cancer Center, Honolulu, HI, USA; g Pediatric Biostatistics Core, Department of Pediatrics, Emory University, Atalanta, GA, USA; h Children’s Minnesota Research Institute, Children’s Minnesota, Minneapolis, MN, USA; i Department of Internal Medicine, University of Texas Southwestern Medical Center, Dallas, TX, USA; j Department of Epidemiology, Tulane University School of Public Health and Tropical Medicine, New Orleans, LA, USA

**Keywords:** CLSA, 2-aminoadipic acid, OBESITY, lipid, Mediation, Mendelian randomization

## 1 Introduction

Obesity is a major risk factor for dyslipidemia and subsequent cardiovascular disease. Excess adiposity induces metabolic disturbances, leading to elevated triglycerides (TG), reduced high-density lipoprotein cholesterol (HDL-C), and increased low-density lipoprotein cholesterol (LDL-C). However, the biochemical intermediates mediating these associations remain incompletely defined. Identifying such mediators can improve mechanistic understanding of obesity-related lipid dysregulation and reveal novel therapeutic targets for CVD prevention.

Two-aminoadipic acid (2-AAA), a catabolic intermediate in the lysine degradation pathway, has emerged as a biomarker of oxidative stress, mitochondrial dysfunction, and metabolic perturbation [1,2], potentially linking obesity to lipid dysregulation. To test this hypothesis, we investigated whether 2-AAA mediates the association between BMI and lipid profiles across two population-based cohorts. Furthermore, given the altered metabolic signaling inherent to obesity, we evaluated effect modification by baseline obesity status and applied Mendelian randomization (MR) to infer directionality.

## 2 Materials and methods

### 2.1 Study population

**Fig. 1:**
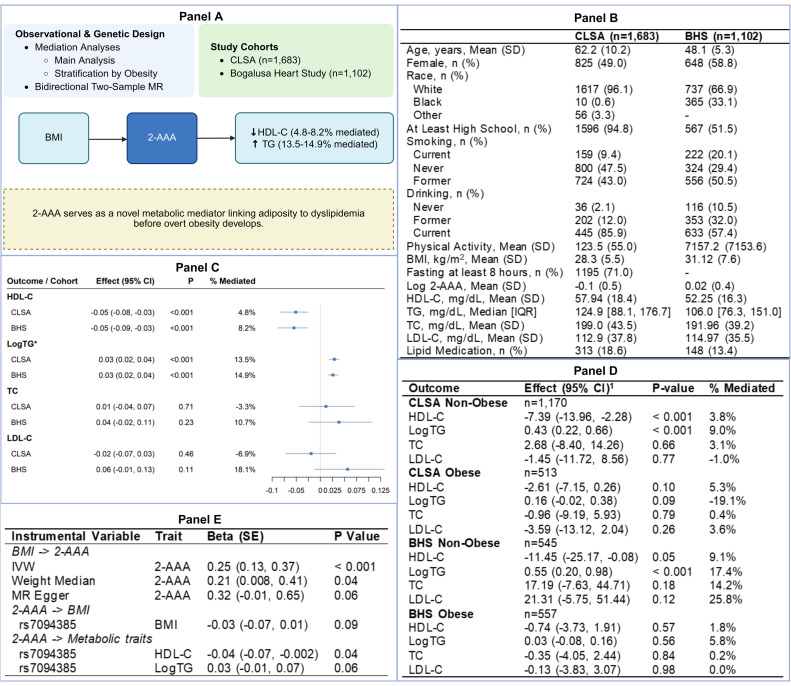
Panel A. Study Design of the Current Study. Panel B. Characteristics of Study Participants. Note: Physical activity was assessed using the International Physical Activity Questionnaire (IPAQ) in BHS and the Physical Activity Scale for the Elderly (PASE) in CLSA. Panel C. Mediation Effects of 2-AAA on BMI-lipid Associations. Note: *Multiplied by 10 for demonstration purpose. Panel D. Mediation Effects of 2-AAA on BMI-lipid Associations Stratified by Obese Status. Note: Mediation effects were estimated by Average Causal Mediation Effect (ACME) calculated using mediation R package. [1] the effects were multiplied by 100 for demonstration purpose. Panel E. Results of Mendelian Randomization Analysis for Directionality Inference. Abbreviations: 2-AAA=2aminoadipic acid, BHS=Bogalusa heart study, BMI=Body mass index, CI=confidence interval, CLSA=Canadian Longitudinal Study on Aging, IVW= inverse variance weighted, HDL-C=High density lipoprotein cholesterol, IQR=Interquartile range, LDL-C=low density lipoprotein cholesterol, SD=Standard deviation, TC=Total cholesterol, TG=Triglyceride.

A multi-stage investigation was conducted (Fig. 1 **Panel A**). First, we performed mediation analysis in a discovery cohort from the Canadian Longitudinal Study on Aging (CLSA) and replicated findings in the Bogalusa Heart Study (BHS). We then performed two-sample MR using summary statistics from large-scale genome-wide association studies (GWAS). This study utilized de-identified, secondary data and is exempt from Institutional Review Board review; original investigators obtained necessary ethical approvals.

*The CLSA* is a prospective cohort of over 50,000 Canadian residents aged 45 to 85 at recruitment, with detailed protocols reported previously[3]. For the present study, our analyses were based on 1683 participants who have available 2-AAA measurements and relevant baseline study variables.

*The BHS* is a longitudinal, community-based cohort of Black (35 %) and White (65 %) residents from Bogalusa, Louisiana. In the 2013–2016 visit cycle, plasma metabolome was profiled among 1261 participants[4]. For this replication analysis, we included 1102 participants with complete measurements for all required study variables.

### 2.2 Exposure (BMI) and outcomes (Lipids)

Height and weight were measured in both cohorts and used to calculate BMI. Lipid measures included total cholesterol (TC), HDL-C, LDL-C, and TG. In both cohorts, TC, HDL-C, and TG were directly assayed from blood samples[4,5]. LDL-C was calculated using the Friedewald equation in BHS and directly assayed in CLSA[5]. TG levels were log-transformed. Fasting status (≥8 h) was recorded, although fasting was only mandatory in the BHS.

### 2.3 Mediator (2-AAA)

Two-AAA were quantified and log-transformed from plasma samples in both cohorts using an identical ultrahigh performance liquid chromatography-tandem mass spectroscopy protocol and quality control procedure[4].

### 2.4 Statistical analysis

Baseline characteristics of the two study cohorts were summarized, with categorical variables presented as frequency (percentage) and continuous variables as mean (standard deviation) or median (interquartile range).

#### 2.4.1 Mediation analysis

R package “mediation” was used to estimate the effect of BMI on lipids transmitted through 2-AAA, and the proportion mediated, adjusting for age, sex, race, education, smoking status, drinking status, physical activity, use of lipid-lowering medication, and fasting status (for CLSA only). Mediation was assessed overall and stratified by obesity (BMI ≥ 30 kg/m²), with robust effects requiring Bonferroni-corrected significance (*p* < 0.0125) in the CLSA discovery cohort alongside nominal significance in the BHS replication cohort.

#### 2.4.2 MR analysis

To assess bidirectional BMI/2-AAA relationships and 2-AAA effects on HDL-C and TG, we conducted two-sample MR using OpenGWAS summary statistics. Genetic instruments (*p* < 5 × 10^–8^) for BMI, HDL-C, and TG were extracted from large European-ancestry GWASs (IEU IDs: ieu-b-40, ieu-b-109, ieu-b-111). For 2-AAA, we selected the *cis*-acting variant rs7094385, a missense mutation at *DHTKD1*, which encodes the rate-limiting enzyme responsible for 2-AAA catabolism. Primary MR analyses utilized the inverse-variance weighted (IVW) method, with weighted median and MR-Egger regressions evaluating pleiotropy and robustness. For 2-AAA exposure models relying on the single rs7094385 SNP, effects were estimated via the Wald ratio, precluding standard sensitivity analyses.

## 3 Results

Characteristics of the participants are presented in Fig. 1 **Panel B**. CLSA participants (*n* = 1683) had a mean age of 62.2 years, were predominantly Whites (96.1 %), and nearly half were females (49.0 %). Most participants had at least a high school education (94.8 %) and were non-smokers (90.5 %).

BHS participants (*n* = 1102) were younger, with a mean age of 48.1 years, and included a higher proportion of females (58.8 %) and Black participants (33.1 %). Only half (51.5 %) had at least high school education, and current smoking was prevalent (20.1 %). Metabolic profiles were generally similar between cohorts, though CLSA participants had a slightly lower mean BMI and a higher rate of lipid-lowering medication use.

After adjusting for covariates, 2-AAA significantly mediated the association of BMI with both HDL-C and TG, but not with TC or LDL-C, in both populations (Fig. 1 **Panel C**). Upon stratification by obesity status, these mediation effects were observed only among non-obese participants (Fig. 1 **Panel D**). In the obese group, no significant mediation effects were observed for any lipid trait.

Two-sample MR (Fig. 1 **Panel E**) revealed that genetically predicted higher BMI significantly increases plasma 2-AAA concentrations, an association robust to horizontal pleiotropy. Conversely, reverse MR indicated no causal effect of 2-AAA on BMI. Regarding downstream lipid effects, genetically predicted 2-AAA was significantly associated with reduced HDL-C and marginally associated with elevated TG levels.

## 4 Discussion

Our study identified a consistent mediating role of 2-AAA in BMI-lipid associations across two independent, demographically diverse populations, with the directionality of these relationships supported by MR analyses. Furthermore, we demonstrated that the mediation effects were much stronger among non-obese individuals, suggesting that the 2-AAA pathway may play a more prominent role during earlier stages of adiposity development. These findings highlight the potential value of targeting the 2-AAA pathway as an early intervention, ideally before the onset of obesity, to prevent downstream dyslipidemia. Such insights may inform stage-specific prevention strategies and the development of personalized therapeutic approaches.

While previous studies have linked 2-AAA to both adipogenesis and metabolic disorders [1,2], our study provided critical missing evidence, at the population level and via MR, that 2-AAA significantly mediates the association between BMI and lipid traits (specifically HDL-C and TG). We further demonstrated this mediation was most relevant before the onset of pronounced obesity, underscoring the importance of early intervention. Targeting 2-AAA pathways may be highly effective in preventing dyslipidemia among individuals who are not yet obese. Recent studies showed shifting to plant-based or seafood diets reduces 2-AAA[6,7]. This effect relies on overall dietary patterns rather than isolated lysine intake, which does not significantly increase 2-AAA[7]. Therefore, our findings support these dietary modifications as an early preventative strategy against metabolic syndrome in at-risk, non-obese populations.

Our study has several limitations. First, the observational part of our study relied on cross-sectional analyses, therefore we could not determine the relationship between long-term changes in 2-AAA and lipids alteration. Second, despite similar findings from previous MR study [1], only one SNP was selected as the instrumental variable for 2-AAA, potentially limited the power of MR analyses. Third, unmeasured confounders driving the observed effects, such as eating behavior, might present. Fourth, the MR analysis relied on GWAS summary statistics of European Ancestry, which could limit the generalizability.

## 5 Conclusion

In two independent cohorts, 2-AAA mediates the causal association between BMI and dyslipidemia (HDL-C and TG), with the strongest effects observed prior to clinical obesity. Future interventional studies among non-obese individuals are warranted to determine if proactively targeting 2-AAA, whether through plant-based dietary modifications or targeted pharmacologic therapies, can successfully disrupt the trajectory toward dyslipidemia.

**Disclaimer:** The opinions expressed in this manuscript are the author's own and do not reflect the views of the Canadian Longitudinal Study on Aging.

During the preparation of this work the author(s) used Gemini in order to polish the language. After using this tool/service, the author(s) reviewed and edited the content as needed and take(s) full responsibility for the content of the published article.

## Data availability

Data are available from the Canadian Longitudinal Study on Aging (www.clsa-elcv.ca) for researchers who meet the criteria for access to de-identified CLSA data.

Data are available upon application to steering committee for researchers to de-identified BHS data.

## CRediT authorship contribution statement

**Ruiyuan Zhang:** Writing – original draft, Visualization, Project administration, Investigation, Formal analysis, Conceptualization. **Yixi Sun:** Writing – review & editing. **Yizhuo Liu:** Data curation. **Tingting Liu:** Writing – review & editing. **Yanling Qi:** Writing – review & editing. **Lang Wu:** Writing – review & editing. **Xiao Sun:** Data curation. **Zhijie Huang:** Data curation. **Yang Pan:** Data curation. **Adrianna Westbrook:** Writing – review & editing. **Shengxu Li:** Resources. **Lydia Bazzano:** Resources, Funding acquisition. **Wei Chen:** Resources. **Jing Chen:** Resources. **Jiang He:** Resources. **Tanika Kelly:** Resources, Funding acquisition. **Changwei Li:** Validation, Supervision, Resources, Methodology, Funding acquisition.

## Declaration of competing interest

The authors declare that they have no known competing financial interests or personal relationships that could have appeared to influence the work reported in this paper.
